# Inhibitory effects of Quercetagetin on the growth, biofilm formation, and virulence factors of bovine mastitis-associated *Escherichia coli*

**DOI:** 10.3389/fmicb.2026.1820626

**Published:** 2026-07-08

**Authors:** Zeyu Wang, Xiaohui Li, Lili Liu, Qing Liu, Ding Guan, Zhen Zhu, Mingze Cao

**Affiliations:** School of Life Sciences and Food Engineering, Hebei University of Engineering, Hebei, China

**Keywords:** biofilm, bovine mastitis, c-di-GMP, *Escherichia coli*, multidrug-resistant, Quercetagetin

## Abstract

**Introduction:**

Bovine mastitis caused by *Escherichia coli* (*E. coli*) poses a significant challenge to the dairy industry, primarily due to biofilm-mediated antibiotic tolerance.

**Methods:**

This study investigated the antibacterial and anti-biofilm activities of Quercetagetin (QG), a natural flavonoid, against highly virulent and multidrug-resistant (MDR) *E. coli* isolates, rigorously screened and selected from a large clinical cohort of 136 bovine mastitis cases.

**Results:**

QG exhibited potent antibacterial activity with Minimum Inhibitory Concentrations (MICs) ranging from 0.5 to 1 mg/mL. Notably, QG not only prevented initial biofilm formation (Minimum Biofilm Inhibitory Concentration; MBIC = 0.5 mg/mL) but also effectively eradicated mature biofilms at a low concentration (Minimum Biofilm Eradication Concentration; MBEC = 1 mg/mL). Scanning electron microscopy (SEM) and confocal laser scanning microscopy (CLSM) confirmed that QG disrupted biofilm architecture, stripped away the extracellular polymeric substance (EPS), and induced bacterial cell lysis. Phenotypic assays revealed that QG significantly (*P* < 0.0001) suppressed swimming, swarming, and twitching motilities and inhibited the synthesis of cellulose and poly-N-acetylglucosamine (PGA). Mechanistically, qRT-PCR analysis demonstrated that QG downregulated the expression of flagellar genes (*fliA*, *fliG*) and the master biofilm regulator *csgD*.

**Discussion:**

Given that the transcription of these genes is positively regulated by the second messenger cyclic dimeric guanosine monophosphate (c-di-GMP), these transcriptional findings support a hypothesis consistent with c-di-GMP pathway involvement, suggesting that QG may impair biofilm integrity by modulating components of this downstream regulatory network. Collectively, these results suggest QG as a promising non-antibiotic candidate compound for controlling *E. coli* mastitis.

## 1 Introduction

Bovine mastitis, predominantly caused by *Escherichia coli*, remains a pervasive threat to the dairy industry, compromising animal welfare and causing substantial economic losses through reduced milk yield and quality (Goetz et al., 2025; Yang et al., 2025). While antibiotics are the standard treatment, their extensive use has accelerated the emergence of multidrug-resistant (MDR) strains and raised concerns regarding drug residues in dairy products (Villarreal et al., 2024; Tvarožková et al., 2025). A critical mechanism underlying this resistance is biofilm formation. The biofilm matrix acts as a physical barrier to antibiotics, while the presence of dormant persister cells and horizontal gene transfer within the biofilm further enhances tolerance and resistance dissemination (Slade et al., 2019; Wu et al., 2025). Consequently, targeting biofilm formation represents a pivotal strategy for controlling MDR *E. coli* mastitis.

The transition from a planktonic to a sessile biofilm lifestyle in *E. coli* is strictly regulated by the second messenger cyclic dimeric guanosine monophosphate (c-di-GMP) (Hidayat et al., 2025). Intracellular c-di-GMP levels, maintained by diguanylate cyclases (DGCs) and phosphodiesterases (PDEs), act as a “master switch” regarding gene expression; high levels activate the transcription factor *csgD*, promoting the synthesis of extracellular matrix components like cellulose and curli fimbriae (Li et al., 2019; Katharios-Lanwermeyer et al., 2021). Therefore, targeting pathways consistent with the disruption of c-di-GMP signaling offers a potential framework to inhibit biofilm initiation and disperse established biofilms.

In the search for sustainable alternatives to antibiotics, plant-derived bioactive molecules and repurposed anti-inflammatory agents have garnered significant attention for their ability to alleviate mastitis and associated inflammatory responses. For instance, recent *in vivo* studies have demonstrated that baicalein, a natural flavonoid, exhibits potent antibacterial and anti-inflammatory properties against methicillin-resistant *Staphylococcus aureus* (MRSA) mastitis, while other agents like diflunisal can attenuate acute inflammatory responses by targeting the NF-κB signaling pathway (Soni et al., 2024). Similarly, quercetin, another well-studied flavonoid, has been shown to inhibit *E. coli* biofilms by interfering with quorum sensing and EPS synthesis (Memariani et al., 2019). Quercetagetin (QG), a flavonol found in *Tagetes erecta*, differs from quercetin structurally by a hydroxyl group substitution and has exhibited notable antioxidant and antimicrobial potential in preliminary studies (Huynh et al., 2024; Yang et al., 2024). Despite these promising traits, the targeted efficacy of QG against *E. coli* biofilm formation and its potential regulatory mechanisms remain critical knowledge gaps.

Given the alarming rise of MDR *E. coli* in dairy farms, identifying the most recalcitrant biofilm-forming strains is crucial. In this study, we hypothesize that QG exerts potent anti-biofilm and antimicrobial effects against these high-risk strains. To ensure clinical relevance, we first conducted an extensive epidemiological screening of 136 clinical mastitis isolates to establish their antibiotic resistance profiles. Subsequently, five representative strains exhibiting the most robust biofilm-producing and MDR phenotypes were selected. The objectives of this work are to investigate the targeted effects of QG on their biofilm formation and virulence factor expression, and to comprehensively explore the underlying transcriptional profiles to propose a consistent mechanistic hypothesis. This research aims to provide a scientific basis for the development of QG-based non-antibiotic therapeutics in the dairy industry.

## 2 Materials and methods

### 2.1 Materials and bacterial strains

Quercetagetin (QG) was purchased from Hebei Handan Chenguang Biotech Group Co., Ltd., and analyzed via gas chromatography-mass spectrometry. QG was dissolved in DMSO, with 1% (v/v) DMSO as the control. The final concentration of DMSO in all assays was maintained below 1% (v/v), which was confirmed to have no significant effect on bacterial growth or biofilm formation compared to the drug-free control. All the solutions were sterilized using 0.22 μm filters prior to use.

The *E. coli* strain ATCC 25922 used for quality control was purchased from the American Type Culture Collection. *E. coli* strains 2, 49, 52, 58, and 113 were isolated from raw milk and preserved at −80°C in our laboratory. All test strains were activated in Luria-Bertani (LB) nutrient agar medium (Qingdao Hope Bio-Technology Co., Ltd.) at 37°C in a constant temperature incubator for 24 h. The strains were cultured in LB broth to the logarithmic phase, and diluted to 1 × 10^6^ CFU/mL as a bacterial suspension for use.

### 2.2 Antimicrobial susceptibility testing (AST)

To determine the antibiotic resistance profiles of the 136 clinical *E. coli* isolates and select representative multidrug-resistant (MDR) strains, the standard Kirby-Bauer disk diffusion method was performed according to the Clinical and Laboratory Standards Institute (CLSI) guidelines. The tested antibiotics included broad-spectrum agents commonly used in veterinary and clinical settings, such as Ampicillin (AMP), Tetracycline (TET), Cefotaxime (CTX), Gentamicin (GEN, Ciprofloxacin (CIP), Enrofloxacin (ENR), and Erythromycin (E), among others. Briefly, bacterial suspensions adjusted to a 1 × 10^6^ CFU/mL were uniformly swabbed onto Mueller-Hinton agar plates (Qingdao Hope Bio-Technology Co., Ltd.). Antibiotic disks were then applied, and the plates were incubated at 37°C for 24 h. The diameters of the zones of inhibition were measured, and the strains were classified as susceptible, intermediate, or resistant based on CLSI interpretive criteria. Strains exhibiting resistance to three or more different classes of antimicrobials were defined as MDR.

### 2.3 Determination of biofilm growth curve

The suspension of *E. coli* strains in LB broth was dispensed into 96-well microtiter plates (200 μL per well), the plates were incubated at 37°C for 96 h. Biofilm growth curve of *E. coli* (1, 2, 4, 8, 12, 24, 48, 72, 96 h) was determined by the crystal violet (CV) method (Katsipis et al., 2025; Ma et al., 2025a; Zeraatpisheh et al., 2025). To quantify the biofilm biomass, the medium was discarded, and wells were rinsed with PBS. The adherent biofilms were fixed with methanol for 15 min and stained with 0.1% (v/v) CV for 5 min. The bound dye was resolubilized with 33% (v/v) glacial acetic acid for 30 min, and the absorbance was measured at 570 nm using a microplate reader (Infinite M Nano, Dicken (Shanghai) Trading Co., Ltd.).

### 2.4 Determination of the minimum inhibitory concentration (MIC) and minimum bactericidal concentration (MBC)

The MICs and MBCs of QG against *E. coli* strains were determined using the broth microdilution method as previously described (Yi et al., 2024) with minor modifications. Briefly, *E. coli* was incubated (37°C, 200 rpm) in Mueller Hinton Broth (MHB, Qingdao Hope Bio-Technology Co., Ltd.) until it reached the exponential growth stage. Serial twofold dilutions of QG were prepared and added to sterile 96-well plates. Finally, 100 μL of the bacterial suspension was added. After culturing at 37°C for 12 h, the MIC was determined to be a well without bacterial growth by measuring OD_600_.

For MBC determination, a 20 μL aliquot of broth from wells representing the MIC and the three next highest concentrations was spread on Mueller Hinton agar (MHA, Qingdao Hope Bio-Technology Co., Ltd.) for colony counting. For each strain, at least three replicates were analyzed, and the modal value was determined.

### 2.5 Determination of the minimum biofilm inhibitory concentration (MBIC) and minimum biofilm eradication concentration (MBEC)

The MBIC and MBEC of QG were determined by a CV method. For MBIC determination, bacterial suspensions (100 μL) were inoculated into 96-well microtiter plates containing 100 μL of serial two-fold dilutions of QG (ranging from 1/4 MIC to 2 MIC). The plates were incubated at 37°C for 48 h. For MBEC determination, biofilms were first allowed to establish by incubating 200 μL of bacterial suspension in 96-well plates at 37°C for 48 h. Following incubation, the planktonic cells were removed by aspirating the medium and washing the wells three times with PBS. Fresh LB broth containing serial twofold dilutions of QG (1/4 MIC to 2 MIC) was then added to the established biofilms, and the plates were incubated for an additional 24 h at 37°C. The MBIC and MBEC are defined as the minimum concentrations of QG that inhibit or eradicate biofilm formation by at least 80%, respectively, compared to the untreated control.

### 2.6 Effect of QG on the motility of *E. coli*

The effects of QG on *E. coli* motility were evaluated using swimming (0.25% agar), swarming (0.5% agar), and twitching (1.0% agar) assays based on a modified protocol by Liu et al. (2024). LB broth supplemented with 0.5% glucose and the respective agar concentrations was autoclaved and cooled to 55°C. QG was added to the media to achieve final concentrations of 1/2 MIC, 1/4 MIC, and 1/8 MIC, while media containing 1% DMSO served as the control.

For swimming and swarming assays, 2 μL of the bacterial suspension was spotted onto the center of the semi-solid agar plates. For the twitching assay, the suspension was stab-inoculated through the agar to the bottom of the petri dish. Swimming plates were incubated at 37°C for 24 h. To facilitate proper colonization, swarming and twitching plates were incubated at room temperature for 1 h prior to incubation at 37°C for 24 h.

Following incubation, the swimming and swarming migration zones were measured directly. For twitching motility, the agar layer was carefully removed, and the diameter of the twitching halo formed at the distinct interface was measured. All assays were performed in triplicate.

### 2.7 Cellulose degradation assay

Cellulose degradation of QG against *E. coli* was determined as described by Rana et al. (2025). Briefly, Congo red agar plates were prepared by autoclaving the medium, cooling to 55°C, and evenly dispensing into petri dishes with three replicates per group. QG was prepared to achieve final concentrations of 0, 1/4 MIC, 1/2 MIC, MIC, and 2 MIC in 20 mL LB broth. Each group was inoculated with 200 μL bacterial suspension, followed by co-incubation for 4 h. Once the agar medium reached a semi-solidified state, 2 μL of the pretreated bacterial suspension was spotted onto the center of each plate. Plates were incubated in a 37°C for 48 h. Cellulose-degrading ability was evaluated by measuring the diameters of the bacterial colonies and the surrounding transparent zones.

### 2.8 Determination of extracellular polymeric substance (EPS) content in biofilms

The inhibitory activity of QG against bacterial biofilm formation was evaluated using a modified ruthenium red staining assay adapted from Zameer et al. (2016). Briefly, bacterial suspensions in LB broth were dispensed in 96-well microtiter plates, with each well receiving 100 μL of bacterial suspension and 100 μL serially twofold-diluted QG (2 MIC-1/4 MIC). The plates were incubated at 37°C for 48 h to form biofilms. After incubation, the culture medium was aspirated, and wells were gently rinsed with PBS to remove non-adherent cells. Following air-drying, 200 μL of the 0.1 % (w/v) ruthenium red staining solution was added to each well, and plates were incubated at 37°C for 1 h. After staining, the ruthenium red solution in each well was transferred to a new sterile 96-well plate to avoid interference from residual biofilm components. Finally, the absorbance value of each well was measured at OD_450_ using a microplate reader.

### 2.9 Scanning electron microscopy (SEM) analysis

The morphological changes of *E. coli* cells within biofilm, treated with QG at different concentrations and untreated controls were imaged via SEM with slight modifications (Ma et al., 2025b). Briefly, for biofilm inhibition, sterile glass coverslips (8 mm diameter) were placed into 24-well plates. Bacterial suspensions were incubated with serially twofold-diluted of QG (MIC-1/4 MIC) at 37°C for 48 h. For biofilm eradication, sterile glass coverslips were placed into 24-well plates with bacterial suspensions at 37°C for 48 h without QG. After mature biofilms were formed, the culture medium was removed, and each well was washed by PBS. Fresh serially twofold-diluted QG (2 MIC-1/2 MIC) was added to wells. The plates were incubated at 37°C for 24 h. After treatment, the coverslips were gently washed three times with PBS. After fixation in 2.5 % glutaraldehyde, the samples were washed with PBS and dehydrated through a graded ethanol series. Ethanol was then replaced with isoamyl acetate. Finally, the specimens were critical-point dried, sputter-coated with gold, and the processed samples were finally observed and analyzed via SEM.

### 2.10 Confocal laser scanning microscopy (CLSM) analysis

To visualize the viability and structural integrity of biofilms, CLSM (LSM800, Zeiss, Jena, Germany) analysis was employed as described by Cho et al. (2023). For biofilm inhibition, *E. coli* cells were treated with QG at aforementioned concentrations at 37°C for 48 h. For biofilm eradication, matured biofilms were treated with QG aforementioned concentrations at 37°C for 24 h. After treatment, the coverslips were gently washed with PBS, then stained with a mixture of SYTO-9 and PI in the dark at 37°C for 15 min. The stained samples were mounted on glass slides and observed via CLSM.

### 2.11 RNA Extraction and quantitative real-time PCR (qRT-PCR) analysis

To elucidate the molecular mechanism underlying the anti-biofilm activity of QG, the transcriptional levels of biofilm and virulence related genes were quantified. Briefly, 200 μL of the bacterial suspension was inoculated into 20 mL of LB broth supplemented with QG to achieve final concentrations of serially two-fold-diluted (2 MIC-1/4 MIC), followed by co-incubation for 12 h. Total RNA was extracted from bacterial pellets using Bacterial Total RNA Extraction kit (Servicebio Biotech, Wuhan, China) following the manufacturer’s protocol. RNA concentration and purity were assessed using a Nano-Drop 2000 spectrophotometer (Thermo Fisher Scientific, Wilmington, DE, United States), and only samples with an A260/A280 ratio between 1.8 and 2.0 were used for subsequent analysis. Reverse transcription was performed to synthesis cDNA using the SweScript All-in-One RT Super Mix (Servicebio Biotech). After reverse transcription, qRT-PCR was conducted on a Bio-Rad CFX96 Real-Time PCR Detection System (Bio-rad CFX Duet, Shanghai, China). The specific primers sequences were listed in Table 1. The relative expression of target genes was calculated using the 2^–ΔΔ^*^CT^* method, with 16S rRNA serving as the internal reference for normalization.

**TABLE 1:** Primer sequences for qRT-PCR.

Gene name	Primer sequences	References
*fliA*	F: CGCTATGCTGGATGAACTTCG	(Buck et al., 2021)
	R: CTAAACGTTCCGCTACCTCAG	
*fliG*	F: GAGCTGACCGAAGTACTGAATG	(Koganitsky et al., 2019)
	R: GGCTTCTTCCTGCTGAGTTT	
*fliM*	F: CAACCTGACCGGCGAATTTA	(Paul et al., 2010)
	R: GCGCCAGTTCTGATCTTCATTA	
*csgA*	F: ATGACGGTTAA ACAGTTCGG	(Sampson et al., 2020)
	R: AGGAGTTAGATGCAGTCTGG	
*csgD*	F: AATCGCTGGCAATTACAGG	(Smith et al., 2017)
	R: CCGCTTCCATCATATCCAG	
*fimH*	F: TGCAGAACGGATAAGCCGTGG	(Zhang et al., 2023)
	R: GCAGTCACCTGCCCTCCGGTA	
*bcsA*	F: GACAGCTACCCGGAAGATAAG	(Acheson et al., 2019)
	R: GAGAAGTCAGCACACGGAATA	
*pgaA*	F: AGGCTTATGTTCGCTGGTATC	(Lin et al., 2020)
	R: TAGTATGGGGTATCGTGTTCTG	
*pgaB*	F: AAACATCCCTCAGGCTAAAGAC	(Lin et al., 2020)
	R: CATTCAGTTGTAATAGGCTCATCC	
*pgaC*	F: GGCGTCTATTTCTGGGTCTATC	(Lin et al., 2020)
	R: GCGGCGTGTATGGTTTCC	
*pgaD*	F: TCTGCTGACGGGTTATTACTG	(Lin et al., 2020)
	R: TATGACTATGTGTGGGTAGATC	
*OmpF*	F: TGCTTATGGTGCCGCTGAC	(Francis et al., 2021)
	R: CGTAGTTCGACTGCCAGGTAG	
*16S rRNA*	F: GATGCATAGCCGACCTGAGA	(Pan et al., 2025)
	R: TGCTCCGTCAGACTTTCGTC	

### 2.12 Statistical analysis

The data analysis and graphical representations were performed with GraphPad Prism 10.1.2 software. For multiple comparisons, *p-*values were analyzed using one-way ANOVA followed by Tukey’s *post-hoc* testing. For time-course analyses (biofilm growth curves), data were evaluated using two-way ANOVA followed by Bonferroni’s multiple comparisons test. A statistically significance was considered as *p* < 0.05, with (*) denoting significant differences relative to Control. All the experiments were performed at least in triplicate, and the data were presented as the mean ± standard deviations (SD). ****(*P* < 0.0001), *** (*P* < 0.001), **(*P* < 0.01), *(*P* < 0.05) and ns (*P* ≥ 0.05).

## 3 Results

### 3.1 High prevalence of MDR and biofilm formation in clinical isolates

Initial screening of the 136 clinical *E. coli* isolates revealed a severe MDR landscape across the sampling cohort. The disk diffusion assays demonstrated widespread resistance to critical veterinary antibiotics. Among the total isolates, 85.3% exhibited resistance to penicillins, and 73% were resistant to tetracyclines, confirming a highly prevalent MDR burden in these bovine mastitis cases.

**FIGURE 1:**
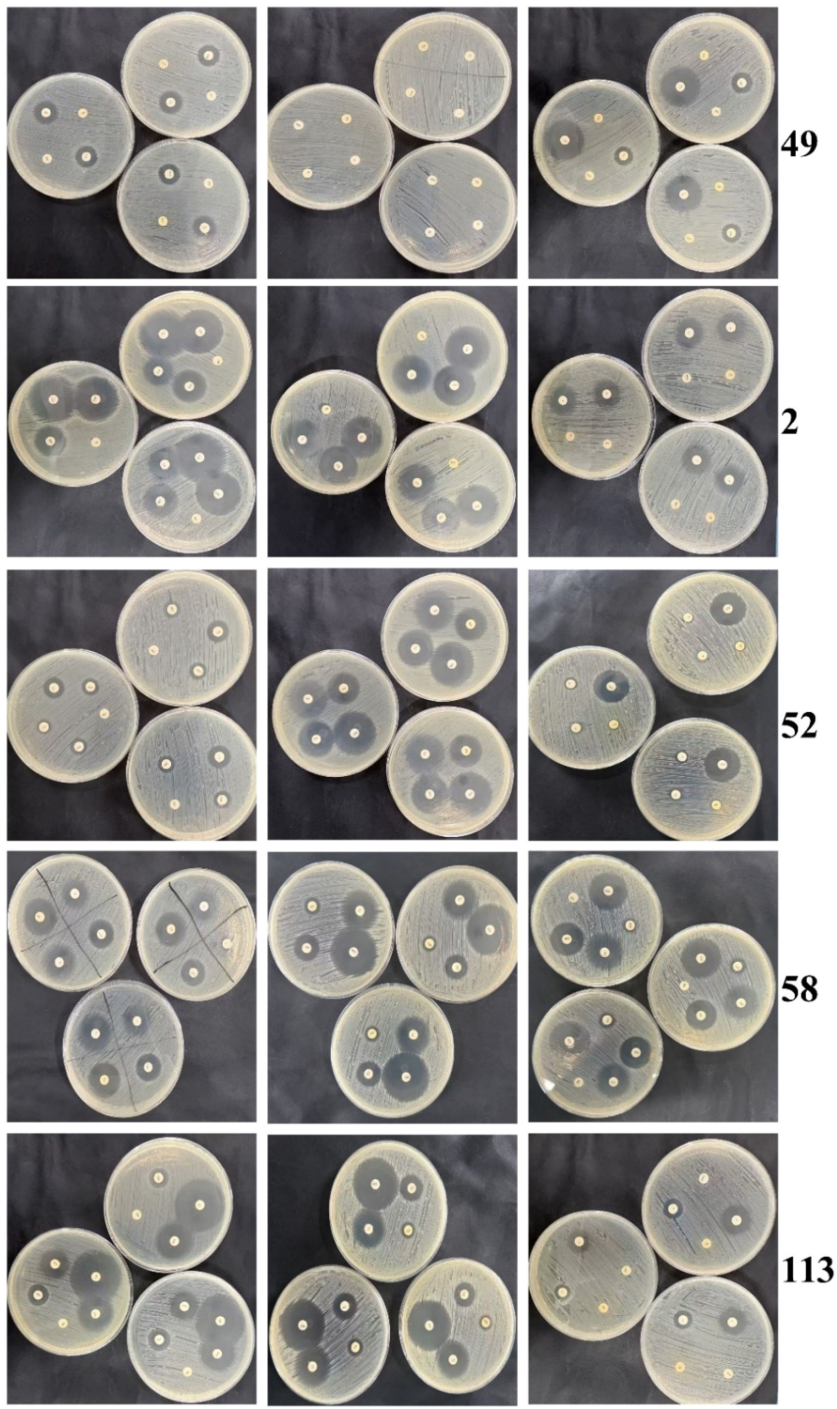
Representative images of the antimicrobial susceptibility testing. To provide clear comparative value, the zone configurations across these 5 representative isolates are benchmarked against the standard CLSI guidelines and the fully susceptible quality control strain ATCC 25922, as systematically quantified and contrasted in Table 2. The lack of distinct inhibition zones around multiple antibiotic disks (e.g., AMP, TET) visually confirms the severe multidrug-resistant (MDR) phenotypes.

From this extensive cohort, five strains (strains 2, 49, 52, 58, and 113) representing diverse extreme phenotypes—exhibiting robust biofilm formation capacities alongside broad-spectrum MDR profiles—were meticulously selected as representative models. As visually illustrated in Figure 1. the disk diffusion plates for these isolates show a stark lack of distinct inhibition zones around multiple antibiotic disks, providing immediate visual confirmation of their highly recalcitrant nature compared to standard clinical breakpoints.

The specific antibiotic susceptibility profiles of these five representative models are systematically quantified in Table 2. All five isolates exhibited resistance to three or more different classes of antimicrobials, strictly classifying them as MDR pathogens. Notably, Strain 49 displayed the most extensive and severe resistance profile, showing complete resistance (R) or intermediate susceptibility (I) to nearly all tested antimicrobial classes, including Clarithromycin, Lincomycin, Ciprofloxacin, Sulfamethoxazole/Trimethoprim, Ampicillin, and Tetracycline. Similarly, the other selected strains demonstrated high rates of resistance to traditional first-line veterinary treatments; for instance, all five strains were resistant to Lincomycin, and a majority were resistant to classic therapies like Ampicillin and Oxytetracycline.

**TABLE 2:** Antibiotic resistance profiles of the five selected representative *E. coli* strains used in this study.

Antimicrobial class	Antibiotic (abbreviation)	Strain 2	Strain 52	Strain 49	Strain 58	Strain 113
Macrolides	Azithromycin (AZI)	S	S	I	I	S
	Clarithromycin (CLR)	S	R	R	R	R
	Erythromycin (E)	I	R	I	I	I
Aminoglycosides	Gentamicin (GEN)	S	R	S	S	I
	Streptomycin (S)	I	R	S	S	R
Lincosamides	Lincomycin (MY)	R	R	R	R	R
Fluoroquinolones	Ciprofloxacin (CIP)	S	I	R	S	S
	Enrofloxacin (ENR)	S	R	I	S	S
Sulfonamides	Sulfamethoxazole/Trimethoprim (SXT)	S	S	R	S	S
Penicillins	Ampicillin (AMP)	R	S	R	S	R
Cephalosporins	Cefotaxime (CTX)	S	S	I	S	S
	Tetracycline (TET)	R	S	R	R	R
Tetracyclines	Oxytetracycline (OT)	R	R	R	S	R

S, Susceptible; I, Intermediate; R, Resistant. The criteria for susceptibility categorization were based on the Clinical and Laboratory Standards Institute (CLSI) guidelines.

### 3.2 Antibacterial efficacy and biofilm growth dynamics

**FIGURE 2:**
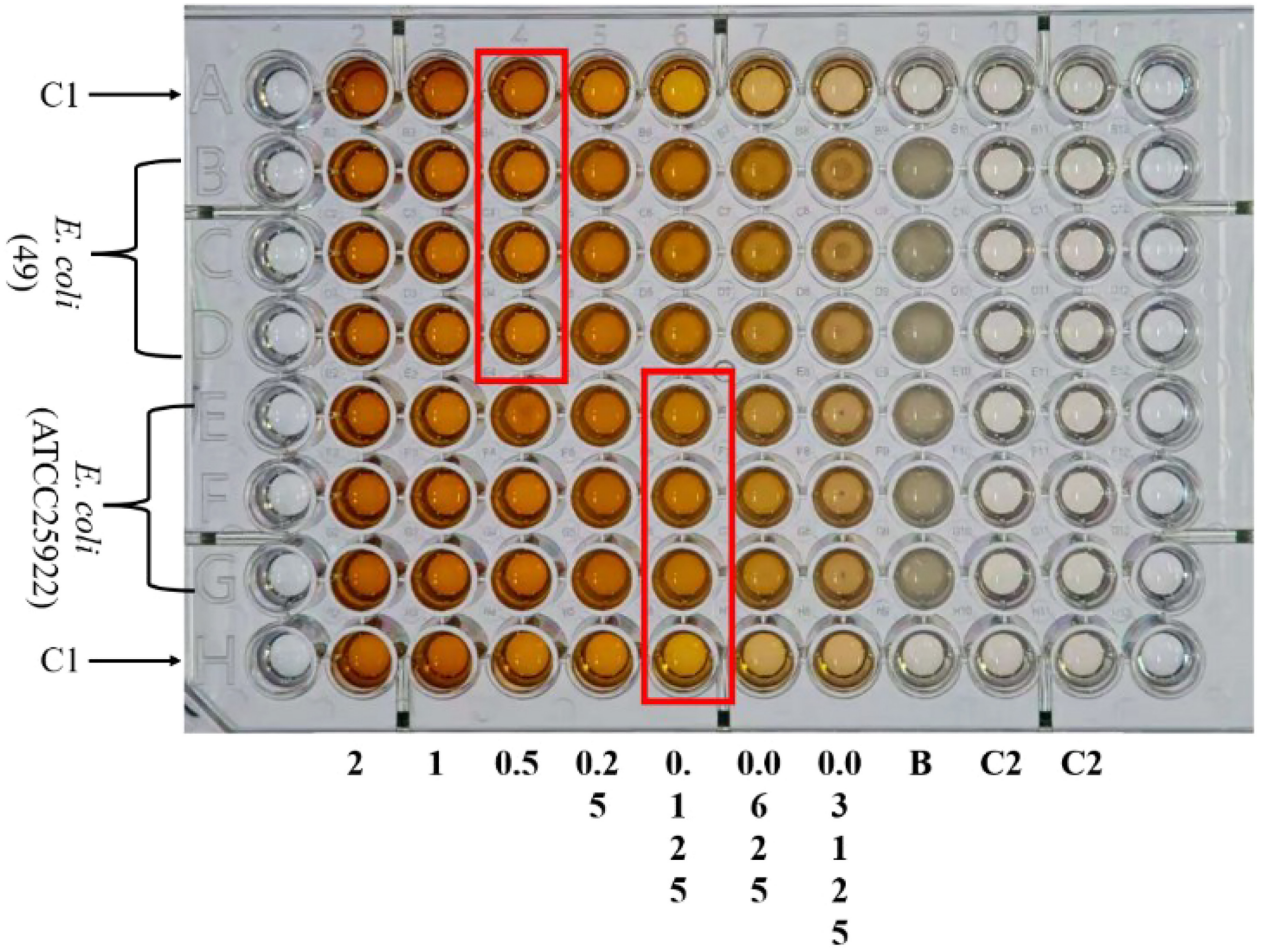
MIC values of QG on *E. coli* 49 and *E. coli* ATCC25922. (C1) The control group without bacteria; (C2) culture medium control; (B) bacterial control group.

We explored the QG antibacterial activities against one reference strain and five clinical isolates of *E. coli*. As shown in Table 3, QG exhibited potent antibacterial activity against all tested *E. coli* strains. For the clinical isolates, the MICs of QG ranged from 0.5 to 1 mg/mL, and the MBCs varied between 1 and 4 mg/mL. Particularly, among the clinical isolates, *E. coli* 49 displayed relative high susceptibility to QG (in Figure 2), with an MIC of 0.5 mg/mL.

**TABLE 3:** MICs and MBCs of QG against six strains of *E. coli* (mg/mL).

*E. coli*	MIC	MBC
ATCC25922	0.125	0.5
2	1	2
49	0.5	1
52	1	4
58	0.5	2
113	1	4

**FIGURE 3:**
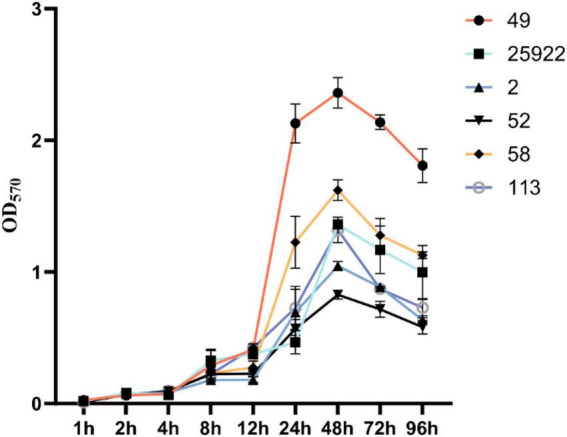
*E. coli* biofilm growth curve.

In addition, we tested all strains for dynamic of biofilm formation (in Figure 3). The results revealed that the strains followed a typical biofilm growth trajectory, with biomass accumulation peaking at 48 h (maturation phase) followed by a decline phase likely due to bacterial detachment. Significant variations in biofilm production were observed across strains. As shown in Figure 3, *E. coli 49* was identified as a strong biofilm producer, exhibiting significantly higher biofilm biomass compared to reference strain ATCC 25922 and other clinical isolates. Based on these profiles, *E. coli* 58 and *E. coli* 113 were classified as moderate producers, and *E. coli* 52 and *E. coli* 2 as weak producers. Therefore, we selected *E. coli* 49 and *E. coli* ATCC25922 as the representative strains for subsequent mechanistic studies.

### 3.3 Effects of QG on the inhibition of biofilm formation and eradication of mature biofilms

**FIGURE 4:**
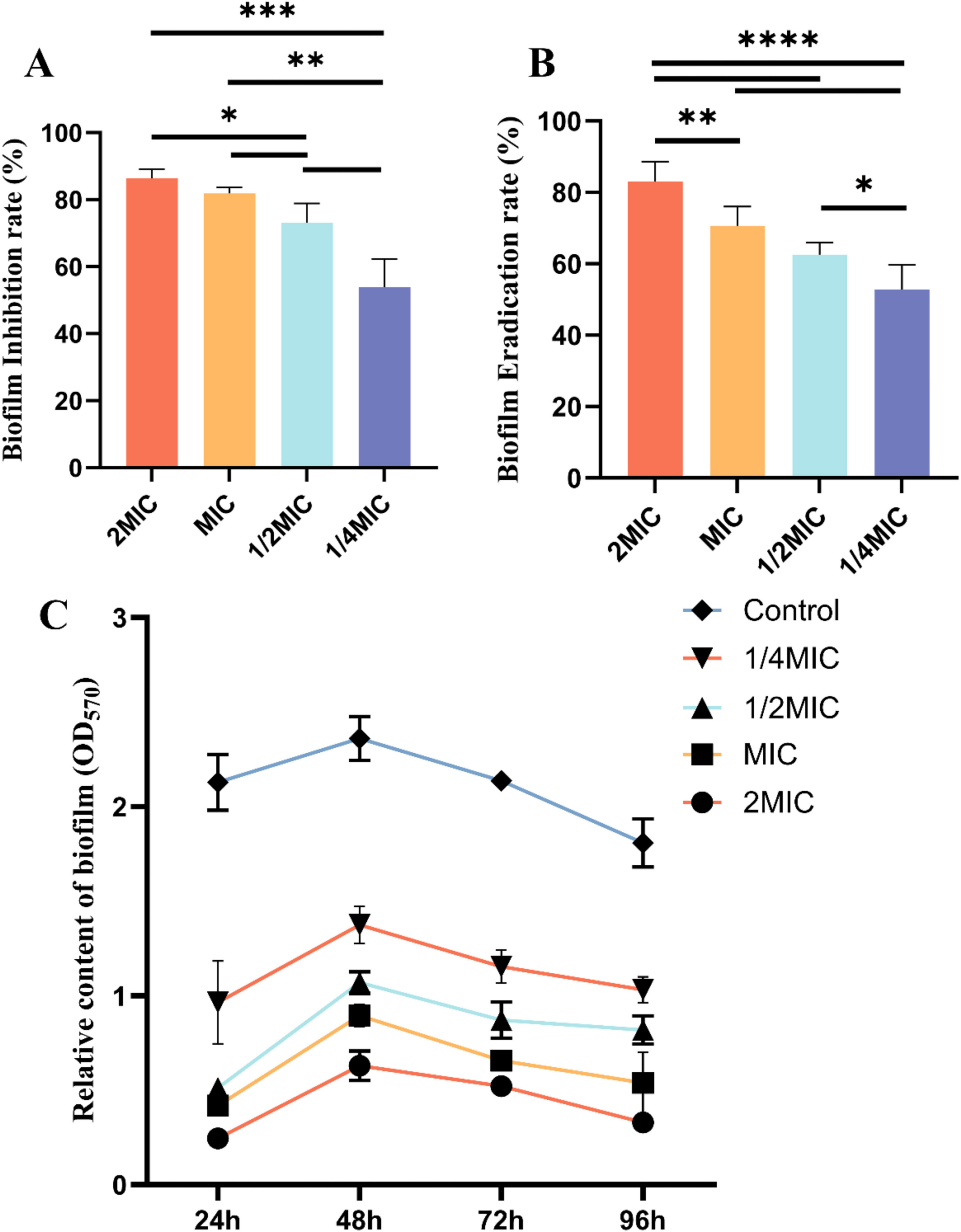
Antibiofilm activity of QG against *E. coli* 49. **(A)** Relative inhibition rates of QG on biofilm formation. **(B)** Relative eradication rates of QG against mature biofilms. **(C)** Time-course analysis of biofilm biomass treated with different concentrations of QG. *P < 0.05, **P < 0.01, ***P < 0.001, and ****P < 0.0001 indicate significant differences between treatment groups or compared to the control.

To comprehensively evaluate the anti-biofilm potential of QG against *E. coli*, we assessed its ability to inhibit biofilm formation and eradicate mature biofilms, as well as its impact on biofilm growth dynamics. As shown in Figure 4A, QG interfered with biofilm development in a significant (*P* < 0.001), dose-dependent manner. Even at a sub-inhibitory concentration of 1/4 MIC, the biofilm inhibition rate reached approximately 50%. As the concentration increased to MIC and 2 MIC, the inhibition rates increased significantly (*P* < 0.001) to over 80%. Consequently, the MBIC was determined to be equivalent to MIC (0.5 mg/mL), indicating that QG effectively prevents the initial colonization and accumulation of biofilms. Eliminating mature biofilms is clinically more challenging than preventing their formation. Therefore, we evaluated the eradication potential of QG on matured biofilms. As illustrated in Figure 4B, QG exhibited potent biofilm-dispersing activity. Treatment with 2 MIC of QG resulted in an eradication rate over 80%. Consequently, the MBEC was determined to be equivalent to 2 MIC (1 mg/mL), indicating that QG can effectively (*P* < 0.0001) penetrate the dense EPS matrix and detach sessile bacteria within mature biofilms. To investigate the sustainability of the inhibitory effect, we monitored biofilm formation over 96 h period under different QG concentrations. As presented in Figure 4C, the control group followed a typical growth trajectory, peaking at 48 h (OD_570_ = 2.3612 ± 0.11). In contrast, QG treatment significantly suppressed biofilm growth at all-time points. Notably, at MIC and 2 MIC, the biofilm biomass was maintained at a remarkably low level, which confirming that QG exerts a durable inhibitory effect on *E. coli* biofilms.

### 3.4 Biofilm architecture and cellular morphology alterations

**FIGURE 5:**
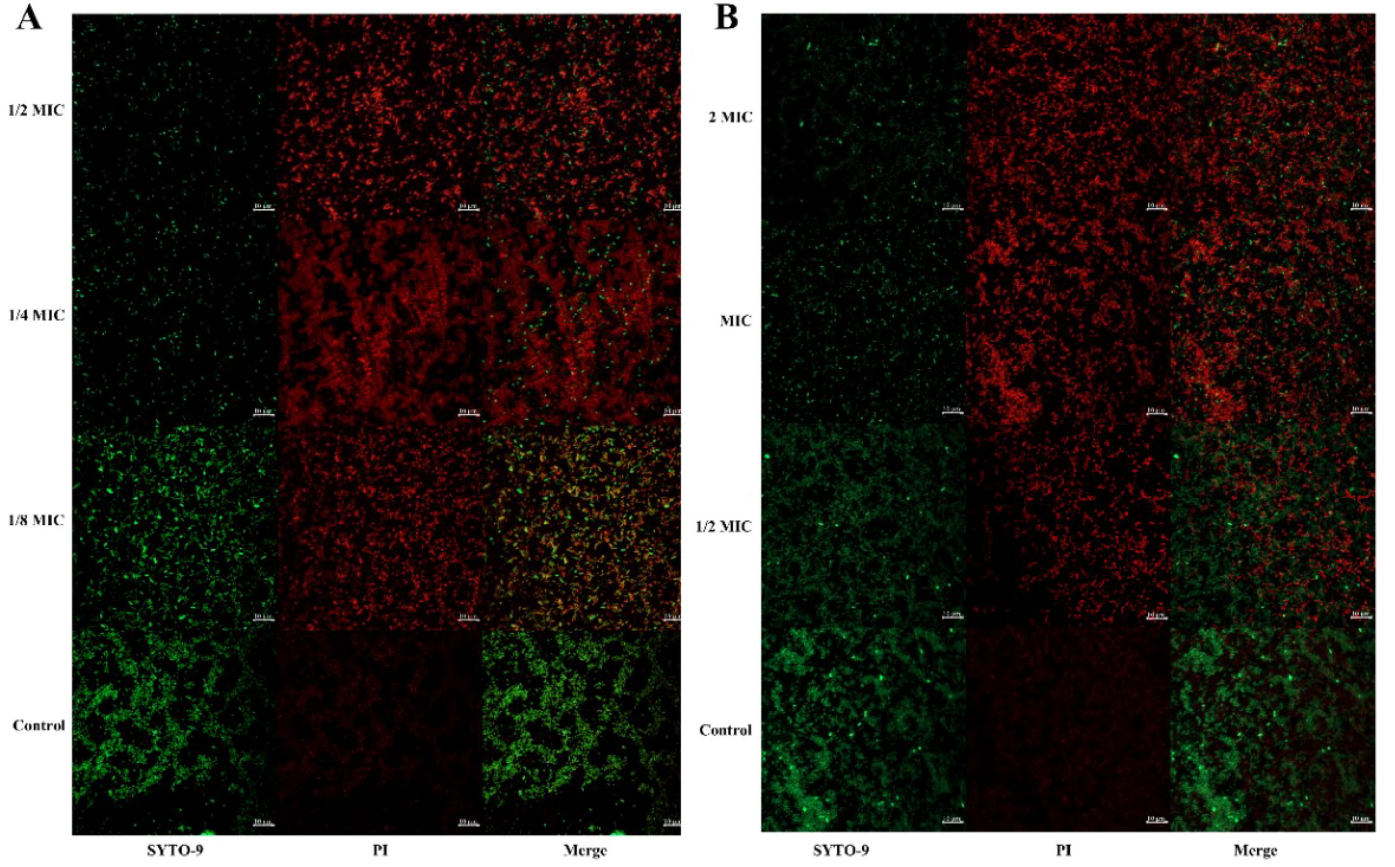
CLSM images of *E. coli* biofilms stained with SYTO-9 (green, live cells) and PI (red, dead cells). **(A)** Biofilm inhibition assay: representative images of biofilms formed in the presence of QG at 1/2 MIC, 1/4 MIC, and 1/8 MIC compared to the control. **(B)** Biofilm eradication assay: representative images of matured biofilms treated with QG at 2 MIC, MIC, and 1/2 MIC compared to the control. Scale bar = 10 μm.

**FIGURE 6:**
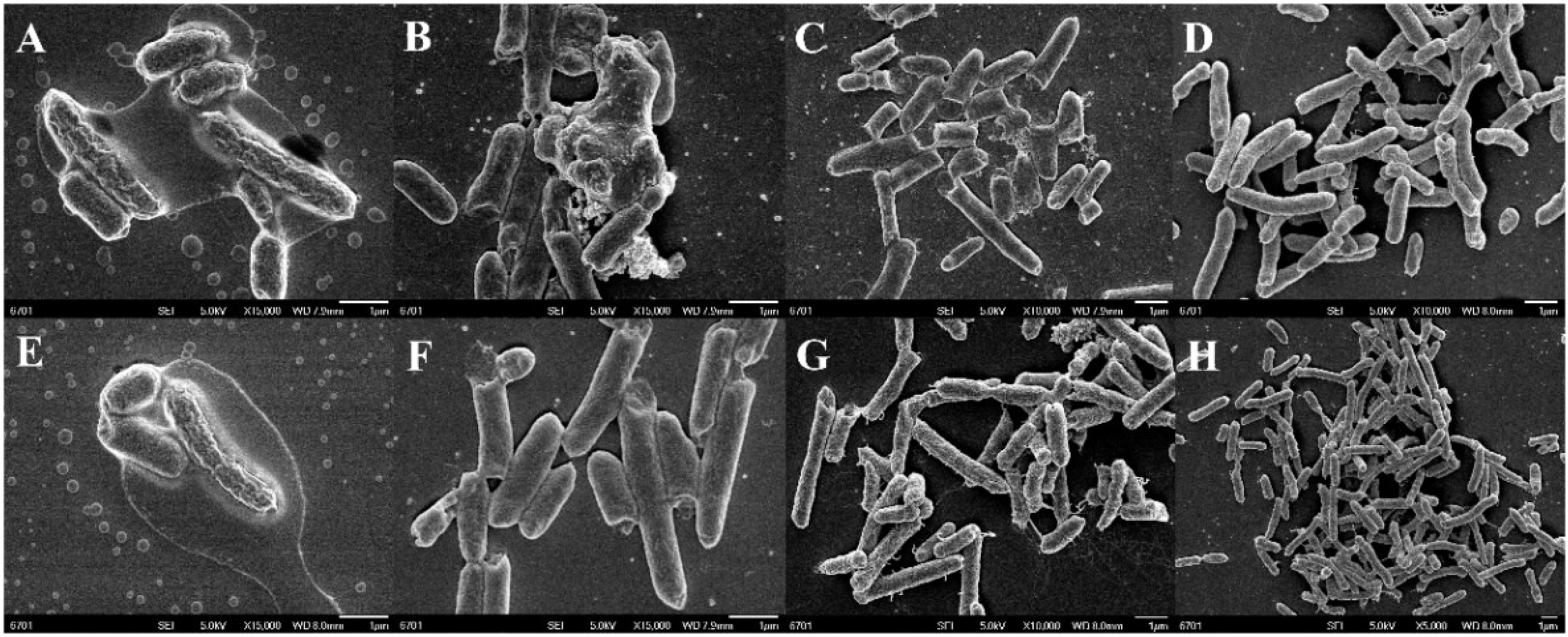
SEM micrographs showing the effect of QG on *E. coli* biofilm morphology. **(A–D)** Biofilm inhibition assay: **(A–C)** Biofilms formed in the presence of 1/2 MIC, 1/4 MIC, 1/8 MIC of QG, showing inhibition of aggregation and EPS synthesis; **(D)** Control; **(E–H)** Biofilm eradication assay: **(E–G)** Biofilms with 2 MIC, MIC, and 1/2 MIC of QG, showing matrix dissolution and cell damage; **(H)** Control. Scale bar = 1 μm.

To visually verify the quantitative findings described above, CLSM and SEM were employed to examine microscopic morphology of *E. coli* biofilms under QG treatment. As shown in Figure 5, CLSM analysis using SYTO-9 and PI staining provided insights into biofilm. The control groups for both assays exhibited a dense, multilayered architecture predominantly emitting green fluorescence, indicating vigorous bacterial growth and intact biofilm structure. In the biofilm inhibition assay (Figure 5A), QG treatment effectively impeded biofilm development. As the concentration increased from 1/8 MIC to 1/2 MIC, the biofilm significantly decreased, green fluorescence degree appearing sparser and thinner compared to the control. In biofilm eradication assay (Figure 5B), QG treatment induced severe disruption of matured biofilms. Treatment with MIC and 2 MIC of QG resulted in a collapsed biofilm structure dominated by red fluorescence, confirming that QG effectively penetrates the matrix and kills sessile bacteria. Moreover, SEM was utilized to observe surface morphology and EPS integrity. As illustrated in Figure 6, the controls (Figures 6D,H) displayed typical untreated biofilm characteristic, with bacterial cells aggregated and encased in a dense EPS. In the biofilm inhibition group (Figures 6A–C), the synthesis of EPS was notably suppressed. Bacterial cells appeared scattered rather than clustered, and the cells surfaces became shriveled and rough. Similarly, in the biofilm eradication group (Figures 6E–H), the dense EPS was effectively stripped away, exposing the bacterial cells. Notably, at 2 MIC (Figure 6E), cells exhibited severe structural damage, including rupture, lysis, and debris formation. These microscopic observations corroborate the MBIC and MBEC results, providing direct evidence of the bactericidal and EPS-degrading mechanisms of QG.

### 3.5 The inhibitory effect of QG on the motility of bacteria

Motility is a critical virulence factor that facilitates the initial attachment of *E. coli* to host mammary tissues and the subsequent formation of biofilms. Therefore, to elucidate the mechanisms by which QG inhibits the initial stages of biofilm formation, we evaluated the effect of QG on swimming, swarming, and twitching motility and the transcriptional levels of key virulence genes.

**FIGURE 7:**
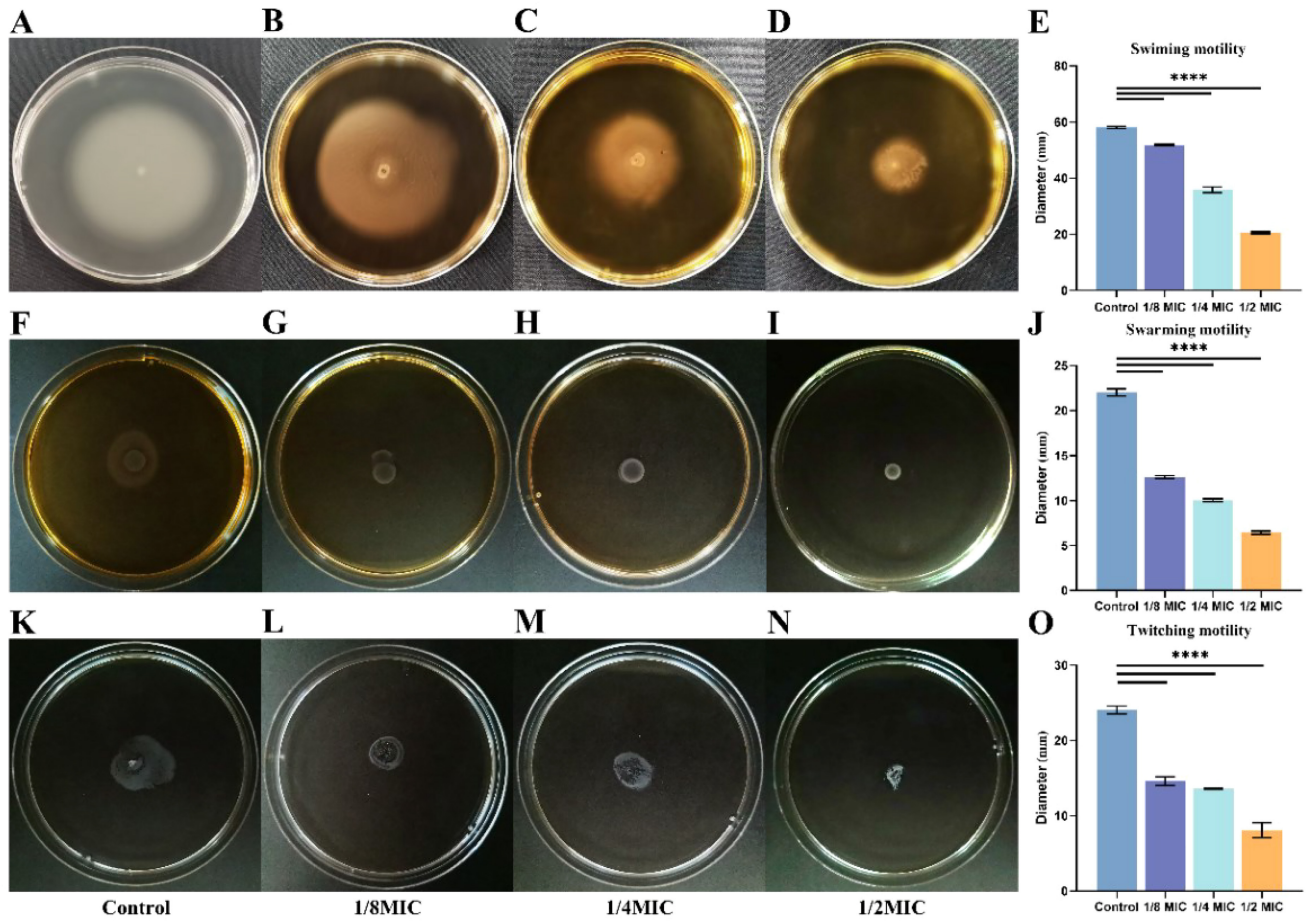
Inhibitory effects of QG on the motility of *E. coli* 49. Representative images of swimming motility **(A–D)**, swarming motility **(F–I)**, and twitching motility **(K–N)**. Quantittive analysis of the diameters of the motility zones for swimming **(E)**, swarming **(J)** and twitching **(O)**. ****Represents a statistically significant difference with *P* < 0.0001 compared to the control group.

As shown in Figure 7, QG significantly impaired the motility of *E. coli* 49. Specifically, for swimming motility, flagella-mediated movement in liquid, the migration diameter significantly (*P* < 0.0001) decreased from 58.06 ± 0.48 mm in the control to 20.57 ± 0.36 mm at 1/2 MIC (Figures 7A–E). Similarly, swarming motility, flagella-mediated movement on surfaces, was markedly suppressed (Figures 7F–J). Notably (*P* < 0.0001), twitching motility, which is driven by type IV pili and is essential for surface “crawling” and biofilm initiation, was also significantly inhibited. At 1/2 MIC, the twitching diameter was reduced by 66.32% compared to the control (Figures 7K–O). Even at a low concentration of 1/8 MIC, a significant inhibitory effect on all three types of the motility was observed (*P* < 0.0001), suggesting that QG effectively interferes with the flagella-mediated movement and pili-mediated surface attachment required for biofilm initiation.

### 3.6 QG suppresses bacterial motility by downregulating flagella and fimbriae related gene expression

**FIGURE 8:**
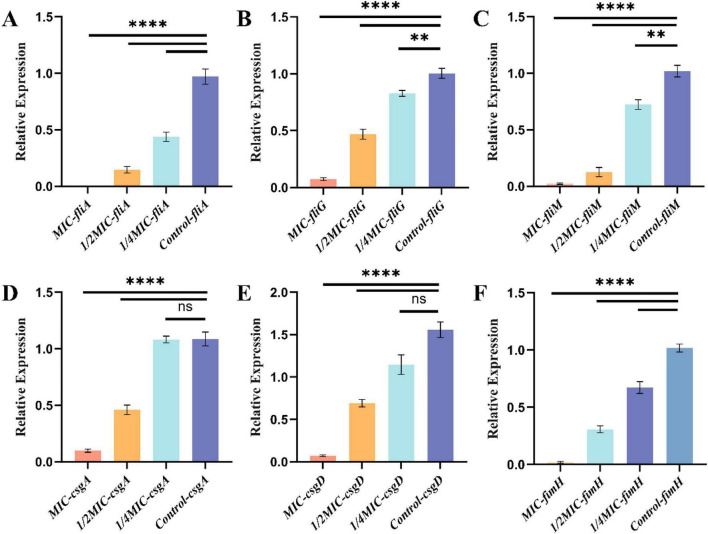
Transcriptional response of flagella- and fimbriae-related genes in *E. coli* 49 to QG treatment. Relative mRNA expression levels of **(A)** *fliA*, **(B)** *fliG*, **(C)** *fliM*, **(D)** *csgA*, **(E)** *csgD*, and **(F)** *fimH* were determined by qRT-PCR. 16S rRNA was used as an internal control. Data are presented as mean ± SD (*n* = 6). ****P < 0.0001, **P < 0.01, and *^ns^P* ≥ 0.05 indicate significant differences compared with the control.

To elucidate the molecular mechanisms underlying these phenotypic changes, we performed qRT-PCR to analyze the transcriptional levels of genes associated with flagellar assembly (*fliA*, *fliG*, *fliM*) and fimbriae synthesis (*csgA*, *csgD*, *fimH*). As illustrated in Figure 8, QG treatment induced a significant downregulation of these target genes in a dose-dependent manner. The expression of flagellar motor-related genes *fliA*, *fliG*, and *fimH* were significantly (*P* < 0.0001) suppressed at MIC and 1/2 MIC (Figures 8A–C), providing a genetic explanation for the reduction in swimming, swarming, and twitching capabilities, aligning with the inhibition of motility. Additionally, QG significantly (*P* < 0.0001) inhibited the expression of genes *csgD*, *csgA*, and *fimH* (Figures 8D–F). Notably (*P* < 0.0001), at MIC, the relative expression of *fimH* and *csgD* was nearly abolished.

### 3.7 QG disrupted biofilm integrity by inhibiting EPS production

**FIGURE 9:**
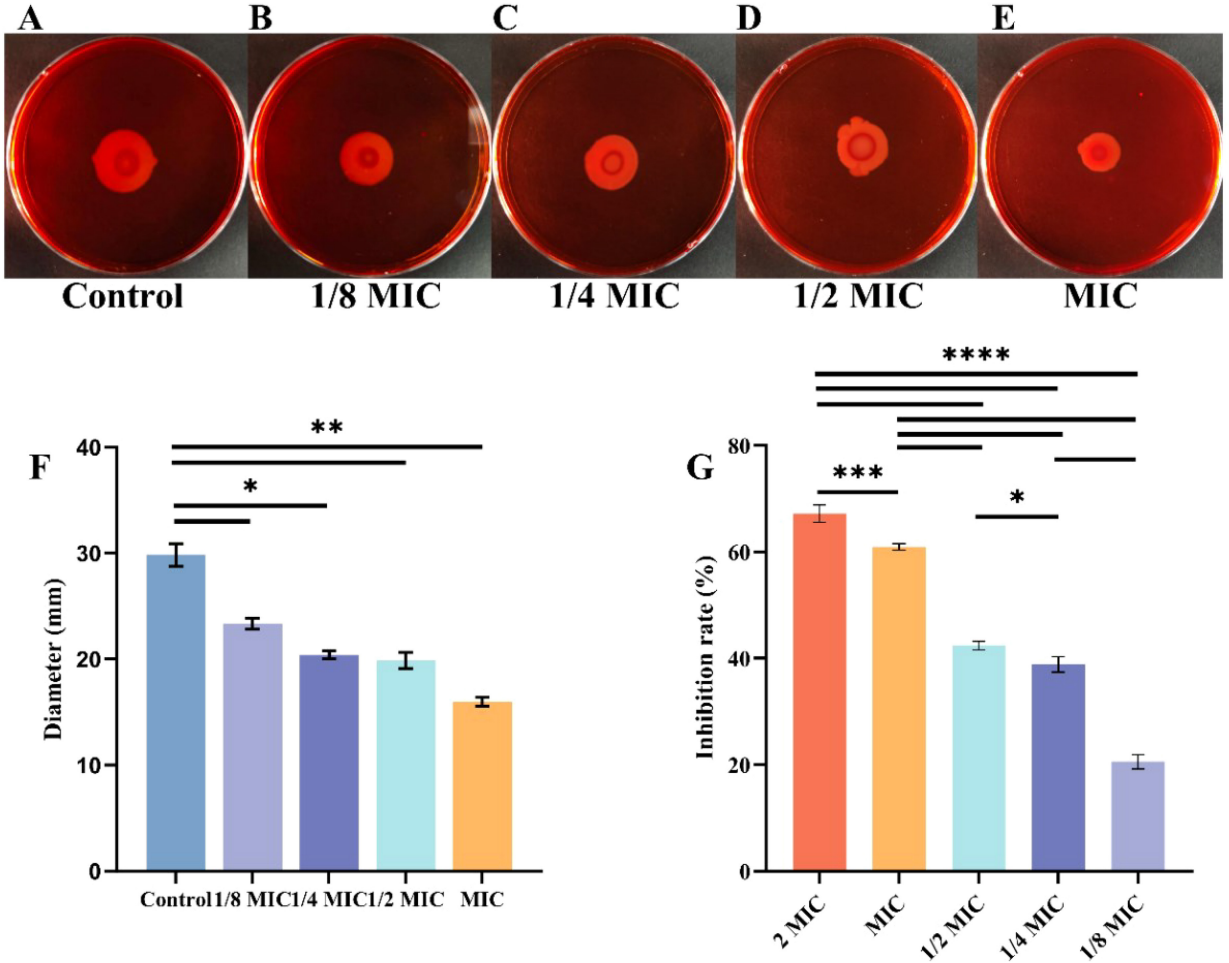
Effect of QG on cellulose production and total EPS content in *E. coli* 49. **(A–E)** Representative photographs of cellulose degradation on Congo Red agar plates treated with various concentrations of QG. **(F)** Quantitative analysis of colony and halo diameters. **(G)** Inhibition rates of total EPS determined by ruthenium red staining. Data are presented as mean ± SD (*n* = 6). ****P < 0.0001, ***P < 0.001, **P < 0.01, and *P < 0.05 indicate significant differences compared with the control.

The EPS primarily composed of cellulose and poly-N-acetylglucosamine (PGA), serve as the fundamental scaffold for biofilms structure integrity and a protective barrier against antibiotics. To elucidate the mechanism of biofilm collapse observed in SEM images, we assessed the effect of QG on matrix component synthesis. To investigate whether QG interferes with biofilm assembly, we performed a cellulose degradation assay using congo red agar. As shown in Figure 9A, the control colonies exhibited a large, dry, and rough morphology with a distinct red halo, and QG treatment resulted in smaller, smoother colonies with significantly reduced halo sizes (Figures 9B–E). The diameter of colonies and their associated transparent zone decreased from 29.84 ± 0.49 mm in the control to 16.01 ± 0.77 mm at the MIC (*P* < 0.01) (Figure 9F), confirmed a dose-dependent reduction in colony diameter. In addition, the total EPS content within the biofilm, quantified via ruthenium red staining, was significantly (*P* < 0.0001) suppressed (Figure 9G). At concentrations of 2 MIC and MIC, the EPS inhibition rate reached 67.2 ± 1% and 60.9 ± 0.7%, indicating that a substantial reduction in biomass of the extracellular.

### 3.8 QG inhibits the expression of EPS-related genes

**FIGURE 10:**
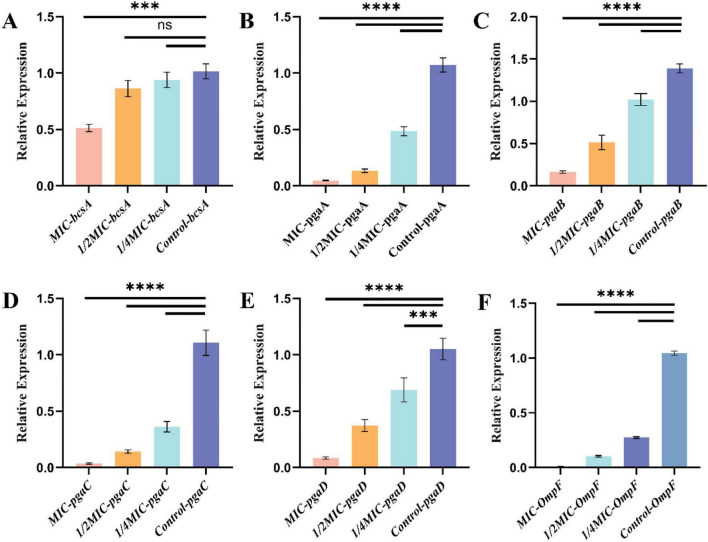
Transcriptional inhibitory effects of QG on biofilm biosynthesis and membrane protein genes in *E. coli* 49. **(A)** *bcsA*; **(B)** *pgaA*; **(C)** *pgaB*; **(D)** *pgaC*; **(E)** *pgaD*); and **(F)** *OmpF* was determined by qRT-PCR. 16S rRNA was used as an internal control. Data are presented as mean ± SD (*n* = 6). ****P < 0.0001, and ***P < 0.001 indicate significant differences relative to the untreated control; *^ns^P* ≥ 0.05 indicate no differences.

To characterize the molecular basis for this matrix reduction, the transcriptional levels of genes responsible for EPS biosynthesis were analyzed in Figure 10. Consistent with the Congo Red results, the expression of *bcsA*, a key gene encoding cellulose synthase, was significantly downregulated in a dose-dependent manner (Figure 10A). Further on, the *pgaABCD* operon is essential for the synthesis and export of the adhesin PGA. The relative expression levels of *pgaA*, *pgaB*, *pgaC*, and *pgaD* were all significantly (*P* < 0.001) reduced compared to the control (Figures 10B–E), aligning with the decrease in total EPS content.

Additionally, the expression of *OmpF*, a major porin protein involved in membrane permeability and biofilm stability, was drastically reduced by QG treatment (*P* < 0.0001) (Figure 10F). The reduction in *OmpF* expression suggests that QG may also limit membrane permeability or hinder the transport of biofilm-related precursors. Collectively, these data confirm that QG compromises the structural integrity of biofilms by transcriptionally repressing the biosynthetic pathways of key biofilm components and modulating membrane porin expression.

## 4 Discussion

Bovine mastitis caused by multidrug-resistant (MDR) *E. coli* represents a persistent and costly challenge to the global dairy industry (Goetz et al., 2025). The recalcitrance of this disease is largely attributed to the ability of *E. coli* to form biofilms, which serve as physical barriers against antibiotics and immune clearance (Elegbeleye and Buys, 2020; Tvarožková et al., 2025). Consequently, the development of non-antibiotic strategies that target bacterial virulence and biofilm architecture has become a research priority (Fidelis et al., 2023; Villarreal et al., 2024). In this study, we demonstrated that QG, a natural flavonol, exhibits potent antibacterial activity and effectively eradicates mature biofilms of clinical mastitis *E. coli* isolates. Furthermore, we elucidated that QG functions by disrupting regulatory network, suppressing flagellar motility, and inhibiting extracellular matrix synthesis.

Our findings align with the growing body of evidence supporting the efficacy of plant-derived natural products against mastitis pathogens. Recent studies have highlighted the potential of various bioactive compounds, such as lycopene (Yang et al., 2025), and naringenin (Yi et al., 2024), in combating *E. coli* infections. Similar to the bacteriocin PFB252 (Katsipis et al., 2025) and enterocin Gr17 (Ma et al., 2025b), QG demonstrated a significant ability to inhibit bacterial growth. Notably, as a flavonoid structurally analogous to quercetin (Huynh et al., 2024), QG shares the broad-spectrum antimicrobial properties observed in quercetin (Cao et al., 2022) and rutin (Almuhanna et al., 2024; Sowmya et al., 2025). Furthermore, our study goes a step further by revealing that QG can eradicate established biofilms at a concentration of only 2 MIC (1 mg/mL).

The suppression of bacterial motility is a critical mechanism by which QG prevents the initiation of biofilm formation. Motility, driven by flagella, is essential for *E. coli* to ascend the teat canal and colonize mammary tissues (Liu et al., 2024). Our results showed that QG significantly downregulated the expression of *fliA*, *fliG*, and *fliM*. Buck et al. (2021) reported that *fliA* is a key regulator of biofilm composition, while Koganitsky et al. (2019) emphasized the role of *fliG* in controlling flagellar motor rotation. The downregulation of these genes explains the observed inhibition of swimming and swarming phenotypes in our study. In addition, the suppression of *fimH* expression, which mediates type I fimbriae adhesion (Zhang et al., 2023), further corroborates the reduced twitching motility and adhesion capability observed. By simultaneously targeting flagella-mediated motility and fimbriae-mediated adhesion, QG effectively immobilizes the bacteria, thereby blocking the early stages of biofilm formation.

Beyond motility, QG severely compromised the structural integrity of the biofilm matrix by targeting the *csgD* regulatory pathway. *csgD* is the key transcription factor that coordinates the expression of curli fimbriae and cellulose (Smith et al., 2017), both of biofilm matrix (Sampson et al., 2020), and highly sensitive to c-di-GMP signaling (Elmanfi et al., 2021). We observed a concurrent downregulation of *csgD*, *csgA*, and *bcsA*, which corresponded to the loss of colony morphology and reduced cellulose production. Furthermore, the downregulation of the *pgaABCD* cluster is of particular clinical significance. Lin et al. (2020) demonstrated that the *pga* locus, responsible for PGA synthesis, plays a pivotal role in conferring antibiotic resistance and facilitating the evolution of resistant strains. By repressing *pgaABCD* operon expression and reducing total EPS content, QG not only destroys the biofilm scaffold but may also sensitize the bacteria to host immune defenses and other antimicrobial agents, offering a potential solution to the challenge of resistance progenitors (Lin et al., 2020).

Our qRT-PCR results (Figures 8, 10) revealed that QG led to a nearly abolished expression of the *pgaABCD* and *csgD* genes. The dramatic suppression points to an interference with the upstream c-di-GMP signaling pathway. In *E. coli*, *csgD* expression is directly stimulated by elevated intracellular c-di-GMP levels (Li et al., 2019). The c-di-GMP signaling system constitutes a complex network involving DGCs and PDEs that modulate bacterial motility, exopolysaccharide secretion, and adhesion capabilities (Elmanfi et al., 2021). In bacteria, low c-di-GMP levels are traditionally associated with active motility, whereas high concentrations promote the production of adhesion factors and extracellular polysaccharides, enhancing biofilm structural integrity (Gong et al., 2023). However, our qRT-PCR results (Figure 8) showed that QG simultaneously down-regulated flagellar genes (*fliA*, *fliG*) and biofilm-promoting genes (*csgD, pgaABCD*). These findings confirm that QG does not merely act by shifting the c-di-GMP balance toward one state, but likely disrupts the global regulatory network governed by c-di-GMP, leading to a comprehensive failure in both surface colonization and movement.

The significant downregulation of *csgD* transcription indicates that QG may act by disrupting cellular c-di-GMP homeostasis (potentially by inhibiting DGC activity or stimulating PDE activity), thereby preventing the accumulation of the threshold c-di-GMP levels required for biofilm maintenance. Targeting global regulatory networks is emerging as a highly effective anti-infective strategy. For example, recent murine mastitis models have shown that alternative therapeutics, such as diflunisal, successfully mitigate *S. aureus*-induced tissue damage and bacterial burden by strongly suppressing the inflammatory NF-κB signaling pathway (Soni et al., 2025). Similarly, our hypothesis aligns with the observed reduction in total EPS content (Figure 9G) and the inhibition of cellulose synthesis (Figures 9A–F). This mechanism of matrix disruption mirrors the effects seen with other antibiofilm agents, such as serrapeptase (Katsipis et al., 2025) and surfactin (Wu et al., 2025), but with the added advantage of QG being a natural dietary flavonoid. While preliminary *in vivo* dietary studies in animal models suggest physiological tolerability (Yang et al., 2024), the lack of direct mammalian cytotoxicity data in our current *in vitro* study warrants careful future evaluation.

It is also noteworthy that QG treatment downregulated *OmpF*, an outer membrane porin. While *OmpF* downregulation is often associated with acquired resistance, in the context of acute flavonoid treatment, it likely reflects a bacterial stress response to membrane perturbation or an attempt to limit the influx of the compound (Francis et al., 2021). This aligns with our SEM observations of cell membrane damage and lysis.

Furthermore, the structural analog quercetin has been reported to bind to several signaling proteins (Tang et al., 2025). It is plausible that the hydroxyl group substitution on the QG molecule allows for even higher affinity with c-di-GMP binding receptors or enzymes. By disrupting this central signaling hub, QG effectively “disarms” *E. coli*, preventing it from sensing surface contacts and initiating the developmental program required for biofilm-mediated bovine mastitis. Future studies utilizing intracellular c-di-GMP quantification assays will further validate QG as a novel c-di-GMP interference agent for sustainable dairy production.

While direct binding and functional knockdown assays of DGCs/PDEs are beyond the scope of this *in vitro* mechanistic exploration, the profound, concurrent transcriptional collapse of the *csgD* master operon, flagellar motor genes (*fliA*, *fliG*), and the *pgaABCD* cluster provides compelling, comprehensive evidence that QG globally destabilizes the c-di-GMP-dependent regulatory network. Furthermore, while *in vitro* studies inherently cannot capture complex host-pathogen interactions, the robust activity of QG against these rigorously screened MDR isolates underscores its clinical potential. Future studies incorporating *in vivo* pharmacokinetic tracking and specific gene-deletion models will build upon this foundational framework.

## 5 Conclusion

In summary, this study identifies QG as a potent botanical therapeutic agent against multidrug-resistant *E. coli* isolated from bovine mastitis. QG exhibits dual functionality: it not only effectively inhibits initial biofilm colonization but, more significantly, eradicates established mature biofilms at a low concentration (1 mg/mL, equivalent to 2 × MIC). This superior eradication capability addresses a critical challenge in clinical mastitis treatment where conventional antibiotics often fail due to biofilm tolerance.

Phenotypically, QG immobilizes bacteria by suppressing swimming, swarming, and twitching motilities and compromises biofilm structural integrity by reducing the synthesis of cellulose and PGA. Mechanistically, these effects are driven by the transcriptional downregulation of the master biofilm regulator (*csgD*) and flagellar genes (*fliA, fliG*). Given that *csgD* expression is positively controlled by c-di-GMP, our transcriptional data support a hypothesis consistent with c-di-GMP interference, suggesting that QG may disrupt the downstream regulatory network essential for the transition from a planktonic to a sessile lifestyle. Collectively, QG represents a potential mechanism-based non-antibiotic candidate compound for controlling bovine mastitis, warranting further *in vivo* validation for clinical application in the dairy industry.

## Data Availability

The original contributions presented in the study are included in the article/supplementary material, further inquiries can be directed to the corresponding authors.
